# Palmitoleic Acid (N-7) Attenuates the Immunometabolic Disturbances Caused by a High-Fat Diet Independently of PPAR***α***

**DOI:** 10.1155/2014/582197

**Published:** 2014-07-24

**Authors:** Camila O. Souza, Alexandre A. S. Teixeira, Edson A. Lima, Helena A. P. Batatinha, Lara M. Gomes, Milena Carvalho-Silva, Isabella T. Mota, Emilio L. Streck, Sandro M. Hirabara, José C. Rosa Neto

**Affiliations:** ^1^Department of Cell and Developmental Biology, University of São Paulo, Avenida Lineu Prestes 1524, 05508-000 São Paulo, SP, Brazil; ^2^Bioenergetic Laboratory, Extremo Sul Catarinense University, 88806-000 Criciuma, SC, Brazil; ^3^Institute of Physical Activity Sciences and Sports, Cruzeiro do Sul University, 01506-000 São Paulo, SP, Brazil

## Abstract

Palmitoleic acid (PMA) has anti-inflammatory and antidiabetic activities. Here we tested whether these effects of PMA on glucose homeostasis and liver inflammation, in mice fed with high-fat diet (HFD), are PPAR-*α* dependent. C57BL6 wild-type (WT) and PPAR-*α*-knockout (KO) mice fed with a standard diet (SD) or HFD for 12 weeks were treated after the 10th week with oleic acid (OLA, 300 mg/kg of b.w.) or PMA 300 mg/kg of b.w. Steatosis induced by HFD was associated with liver inflammation only in the KO mice, as shown by the increased hepatic levels of IL1-beta, IL-12, and TNF-*α*; however, the HFD increased the expression of TLR4 and decreased the expression of IL1-Ra in both genotypes. Treatment with palmitoleate markedly attenuated the insulin resistance induced by the HFD, increased glucose uptake and incorporation into muscle in vitro, reduced the serum levels of AST in WT mice, decreased the hepatic levels of IL1-beta and IL-12 in KO mice, reduced the expression of TLR-4 and increased the expression of IL-1Ra in WT mice, and reduced the phosphorylation of NF *𝜅*B (p65) in the livers of KO mice. We conclude that palmitoleate attenuates diet-induced insulin resistance, liver inflammation, and damage through mechanisms that do not depend on PPAR-*α*.

## 1. Introduction

Chronic positive energy balance through inadequate dietary habits and a sedentary life style leads to an excessive accumulation of body fat, known as obesity, the prevalence of which is increasing alarmingly worldwide [1]. Obese individuals have a greater risk for the development of many chronic and highly incident diseases such as type 2 diabetes, dislipidemia, hepatic steatosis, and some types of cancer [2–6].

Among these diseases, nonalcoholic fatty liver disease (NAFLD), which is defined as excessive hepatic lipid accumulation, is one of the most common comorbidities associated with obesity and insulin resistance. Along with the obesity epidemic, the incidence of NAFLD is growing worldwide; NAFLD now affects an estimated 20–30% of the population of Western countries [1]. If not treated, hepatocellular steatosis may progress to more severe diseases such as nonalcoholic steatohepatitis (NASH), liver cirrhosis, and hepatocellular carcinoma [7, 8].

Consistent with the key role of the liver in the regulation of glucose metabolism, excessive fat accumulation in the liver promotes a local inflammatory process that is frequently associated with the development of tissue insulin resistance and major changes in glucose homeostasis. More specifically, hepatic steatosis and inflammation markedly impair the ability of insulin to inhibit liver glucose production, leading to hyperglycemia and hyperinsulinemia [9, 10]. Therefore, strategies to counteract hepatic steatosis, inflammation, and increased hepatic glucose production are crucial to the prevention and treatment of chronic metabolic diseases.

Palmitoleic acid (16:1n7), a monounsaturated fatty acid (n-7) of 16 carbons that is produced in adipose tissue, has been shown to have important metabolic activities that improve whole body glucose homeostasis and insulin sensitivity [11]. Indeed, palmitoleic acids were shown to increase insulin-stimulated glucose uptake by the skeletal muscles [11] and to reduce liver steatosis, inflammation, and insulin resistance, thus attenuating high-fat diet-induced hepatic glucose production [12].

Mechanistically, palmitoleic acid reduces hepatic steatosis by inhibiting the expression of sterol regulatory element binding protein-1 (SREBP1), a transcription factor that is involved in the regulation of many enzymes involved in lipid synthesis, including fatty acid synthase (FAS) and stearoyl-CoA desaturase 1 (SCD1) [13]. Very recently, it was demonstrated that palmitoleic acid is a positive modulator of white adipose lipolysis through a mechanism that involves an increase in the content of the lipase adipose triglyceride lipase (ATGL) and requires the activation of nuclear receptor PPAR*α* [14]. Peroxisome proliferator activated receptor *α* (PPAR*α*), a transcription factor that is primarily expressed in the liver and modulates the transcription of enzymes related to *β*-oxidation, promoting the oxidation of fatty acids and an overall reduction in the deposition of ectopic triacylglycerol [15]. Furthermore, PPAR*α* was also shown to suppress the expression of proinflammatory genes, primarily by inactivating the master proinflammatory transcription factor NF*κ*B and thus reducing the production of cytokines and tissue inflammation [16].

Based on these findings, we tested the hypothesis that palmitoleic acid attenuates obesity-associated hepatic steatosis and inflammation by activating the nuclear receptor PPAR*α*. For this, wild-type and PPAR*α*-knockout mice were fed a high-fat diet, either untreated or treated with palmitoleic acid, and evaluated for hepatic steatosis and inflammation and whole body glucose homeostasis.

## 2. Materials and Methods

### 2.1. Animal Procedure

Male C57BL/6J wild-type (WT) and PPAR*α*-knockout (KO) mice were obtained from the Jackson Laboratory and maintained on a 12:12 h light-dark cycle (lights on at 06:00). Beginning at 10 to 12 weeks of age, the mice were fed a high-fat diet (HFD, 59% of calories from fat, 15% from proteins, and 26% from carbohydrates) [17] or a low-fat diet (LFD, 9% of calories from fat, 15% from protein, and 76% from carbohydrate) [17] for 12 weeks. In the last 2 weeks, the HFD-fed mice were treated with oleic acid (300 mg/kg of body weight) or palmitoleic acid (300 mg/kg of body weight) daily by oral gavage. The doses and treatment regimen were based on previous studies [13, 14]. During the feeding/treatment period, the body weight and food intake of the mice were evaluated weekly. After 12 weeks, the mice were fasted for 4 hr and then sacrificed for the collection of blood and tissue samples. The epididymal, mesenteric, and retroperitoneal adipose tissues were dissected and weighed, and the total weight of these tissues was represented as adipose tissue index. The liver was weighed and stored for the further analysis of RNA and protein.

### 2.2. Analytical Procedures

Plasma total cholesterol, HDL cholesterol, triacylglycerol levels, and alanine aminotransferase activity were determined by enzymatic methods (Labtest, Lagoa Santa, MG, Brazil). The LDL levels were estimated using the Friedewald equation [18].

### 2.3. Histological Analyses

Small pieces of liver tissue were fixed with paraformaldehyde (10%), embedded in paraffin, and serially cross-sectioned. The slides were stained with hematoxylin and eosin to analyze steatosis [19].

### 2.4. Assessment of Triacylglycerol Levels in the Liver

Lipids were extracted from the livers with chloroform-methanol, as described by Folch et al. [20]. Tissue triacylglycerol levels in the lipid extract were determined by enzymatic assays (Labtest, Lagoa Santa, MG, Brazil).

### 2.5. Insulin and Glucose Tolerance Tests

Mice fasted for 4 hr received an intraperitoneal injection of insulin (1 U/kg body weight) or D-glucose (2 g/kg body weight). For the insulin tolerance tests, blood samples (5 *μ*L) were collected from the tail vein before and at 10, 20, 30, and 40 min after the bolus insulin injection. The constant for plasma glucose clearance (KITT) was calculated by linear regression of the glycemic levels measured between 5 and 30 minutes after insulin injection; this is the interval in which the glucose linear decay phase occurs [21]. Similarly, for the glucose tolerance tests, blood samples were collected from the tail vein before and at 15, 30, 60, 90, and 120 min after the glucose bolus injection [22]. The differences in glycemia before and during glucose administration were used to calculate the areas under the curve (AUC). The levels of plasma glucose were measured using an Accu-Chek Performa glucometer (ROCHE, São Paulo, SP, Brazil).

### 2.6. Insulin Response in Isolated Soleus Muscles

Soleus muscles from euthanized WT and KO mice were carefully isolated, weighed (8–10 mg), and attached to stainless steel clips to maintain resting tension. The muscles were preincubated in Krebs-Ringer bicarbonate buffer (KRBB) containing 5.69 mM glucose and 1% bovine serum albumin (BSA), pH 7.4, and pregassed (95% O_2_, 5% CO_2_) with agitation (100 oscillations/min). After these procedures, the muscles were transferred to fresh vials containing the same buffer containing 0.3 *μ*Ci/mL D-[U-^14^C]-glucose and 0.2o *μ*Ci/mL 2-deoxy-D-[2,6-^3^H]-glucose in the presence or absence of 7 nM insulin. After the incubation period, the samples were processed to measure the uptake of 2-deoxy-D-[2,6-^3^H]-glucose, the incorporation of D-[^14^C]-glucose, the synthesis of [^14^C]-glycogen, and the decarboxylation of D-[^14^C]-glucose, according to the methods described by Challiss et al. [23], Espinal et al. [24], and Leighton et al. [25].

### 2.7. Enzymatic Assays

Livers were homogenized (1 : 10, w/v) in SETH buffer, pH 7.4 (250 mM sucrose, 2 mM EDTA, 10 mM Trizma base, and 50 IU/mL heparin). The homogenates were centrifuged, and the supernatants were stored at −70°C for use in enzyme activity determination. The protein content of the homogenates was determined by the method described by Lowry and colleagues [26]. The activity of citrate synthase was assayed according to the method described by Srere, and the reaction was initiated by adding oxaloacetate (0.2 mM) to a mixture containing Tris (100 mM, pH 8.0), acetyl CoA (0.1 mM), dithiobis-2-nitrobenzoic acid (0.1 mM), Triton X-100 (0.1%), and 2 to 4 *μ*g of supernatant protein and monitored at 412 nm for 3 min at 25°C [27]. The activity of succinate dehydrogenase was measured according to the method of Fischer and colleagues [28] as a decrease in the absorbance of the reactions at 600 nm caused by the reduction of 2,6-di-chloro-indophenol in the presence of phenazine methosulfate. The activity of malate dehydrogenase was measured as described by Kitto et al. [29]. The activity of NADH dehydrogenase (complex I) was evaluated according to Cassina and Radi [30], and the activity of the succinate: cytochrome c oxidoreductase (complex III) was determined using the method described by Fischer and colleagues [28].

### 2.8. Enzyme-Linked Immunosorbent Assay (ELISA)

Liver tissue samples (80–100 mg) were carefully homogenized in RIPA buffer (0.625% Nonidet P-40, 0.625% sodium deoxycholate, 6.25 mM sodium phosphate, and 1 mM ethylenediaminetetraacetic acid at pH 7.4) containing 10 *μ*g/mL protease inhibitor cocktail (Sigma-Aldrich, St. Louis, MO, USA). The homogenates were centrifuged, the supernatant was utilized to determinate the protein concentration via Bradford assays (Bio-Rad, Hercules, CA, USA), and the protein levels of IL-1*β*, IL-8, IL-12, and TNF-*α* were measured by ELISA (DuoSet ELISA, R&D Systems, Minneapolis, MN, USA). For IL-1*β*, IL-8, IL-12, and TNF-*α*, the assay sensitivity was 5.0 pg/mL in a range of 31.2 to 2,000 pg/mL.

### 2.9. RNA Isolation, Reverse Transcription, and Real-Time PCR

The expression of hepatic genes related to fatty acid synthesis (ACC) and some factors involved in inflammation (IL-1Ra, TLR4 and TNF-*α*) was assessed by qRT-PCR with SYBR Green marker. For this, total RNA was extracted as described by Chomczynski and Sacchi [31] and quantified in a spectrophotometer (260 nm), and cDNA was synthesized from the total RNA using reverse transcriptase. The sequences of the primers are shown in Table 1; gene expression was quantified by the comparative method using the expression of GAPDH as standard [32].

### 2.10. Western Blotting

The livers were carefully homogenized in extraction buffer containing protease and phosphatase inhibitors. After proper centrifugation, the protein concentrations were determined by Bradford assay (Bio-Rad, Hercules, CA, USA). Aliquots of each sample with the same concentration of total protein (25 g) were then diluted in Laemmli buffer, subjected to electrophoresis on SDS-polyacrylamide gel electrophoresis (SDS-PAGE), and transferred from the gel to a nitrocellulose membrane. These membranes were incubated with antibodies against Toll-like receptor 4 (1 : 500), Phospho-NF*κ*B p65 (Ser536) (1 : 500) (Cell Signaling Technologies, USA), or *β*-tubulin (1 : 1.000) (Santa Cruz Biotechnology, USA) and then incubated with an anti-IgG antibody conjugated with peroxidase. After the incubations, these membranes were incubated with the peroxidase substrate (ECL kit, Biorad, USA) and exposed to X-ray film.

### 2.11. Statistical Methods

The data are presented as mean ± SEM (standard error of the mean) and analyzed by one-way analysis of variance (one-way ANOVA) followed by Bonferroni posttests. Analyses were performed using GraphPad Prism 5.0 software. Differences were considered significant when *P* < 0.05.

## 3. Results

We did not observe any differences in the variables analyzed between WT and KO mice fed with the control SD diet (see Supplementary Table of the Supplementary Material available online at http://dx.doi.org/10.1155/2014/582197); therefore, for the sake of objectivity, we are showing only the data for WT mice fed with the standard control diet.

As expected, the HFD markedly increased the body weight gain of WT and KO mice, and this effect was of lower magnitude in the KO mice (40% versus 10%, resp.) (Table 1). This increased body weight gain induced by the HFD was associated with a marked increase in mouse adiposity, as evidenced by the greater sum of the masses of the major adipose deposits (epididymal, retroperitoneal, and inguinal, see adipose tissue index, Table 2). In contrast to the white adipose tissue, however, HFD feeding did not affect the mass of the brown adipose tissue in any of the groups tested (Table 2). The HFD significantly increased total cholesterol, and in KO mice, the estimated levels of LDL were higher (Table 2). The fasting glucose levels were higher in the animals that were subjected to the HFD, but the KO mice had lower glycemia than the WT mice (Table 2). Treatment with palmitoleic acid (PMA) did not alter the body weight, the weights of the white and brown adipose tissues, or the plasma lipid profile of either group, but treatment with palmitoleic acid reduced the fasting glucose levels in both WT and KO mice that were subjected to the HFD (Table 2).

**Figure 1:**
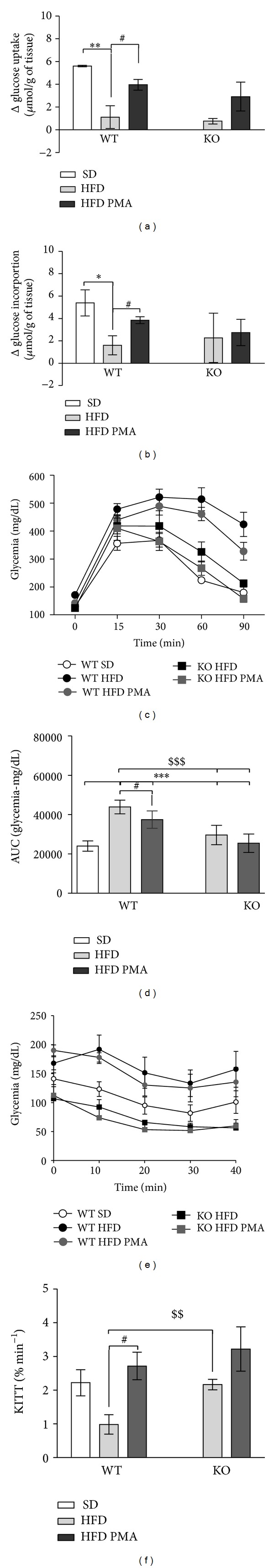
Delta (Δ) of glucose uptake (a) and incorporation (b), variation in glycemia in the glucose tolerance test (c), and respective area under curve (AUC) (d). Variation in glycemia in the insulin tolerance test (e). Respective constants for glucose clearance (f) of WT mice fed with a standard diet (SD) or WT and PPAR*α*-knockout (KO) mice subjected to a high-fat diet and treated with oleic acid (HFD) or palmitoleic acid (HFD PMA). The data are presented as mean ± SEM. *P < 0.05, **P < 0.01, and ***P< 0.001 versus WT SD; ^#^ *P* < 0.05 HFD versus HFD PMA; and ^$$^ *P* < 0.01 and ^$$$^ *P* < 0.001 KO versus the respective WT control (one-way ANOVA followed by Bonferroni correction).

Our results showed that independent of the mice genotype, HFD feeding reduced the peripheral responsiveness to insulin. WT and KO mice subjected to the HFD had a reduced glucose uptake, but WT mice had a reduced incorporation of glucose by insulin stimulation (Figures 1(a) and 1(b)). When compared to SD-fed WT mice, both WT and KO mice subjected to the HFD had a greater increase in glycemia in a glucose tolerance test (GTT) (Figure 1(c)), and this metric could be confirmed by the increase in the AUC (Figure 1(d)); however, compared to HFD-fed WT mice, the HFD-fed KO mice presented a higher glucose tolerance. In addition, the HFD impaired the response to insulin in WT mice but not in KO mice (Figure 1(e)), who had a better responsiveness to insulin as shown by the diminished KITT (Figure 1(f)). Additionally, we observed that independent of the mouse genotype, palmitoleate markedly attenuated the insulin resistance induced by HFD, as evidenced by the increased glucose uptake (Figure 1(a)) and by the better response to insulin (Figure 1(h)) in both groups. The palmitoleate also increased the incorporation of glucose into muscle in vitro (Figure 1(b)) and improved the tolerance to glucose (Figure 1(d)) in WT mice but not in KO mice.

**Figure 2:**
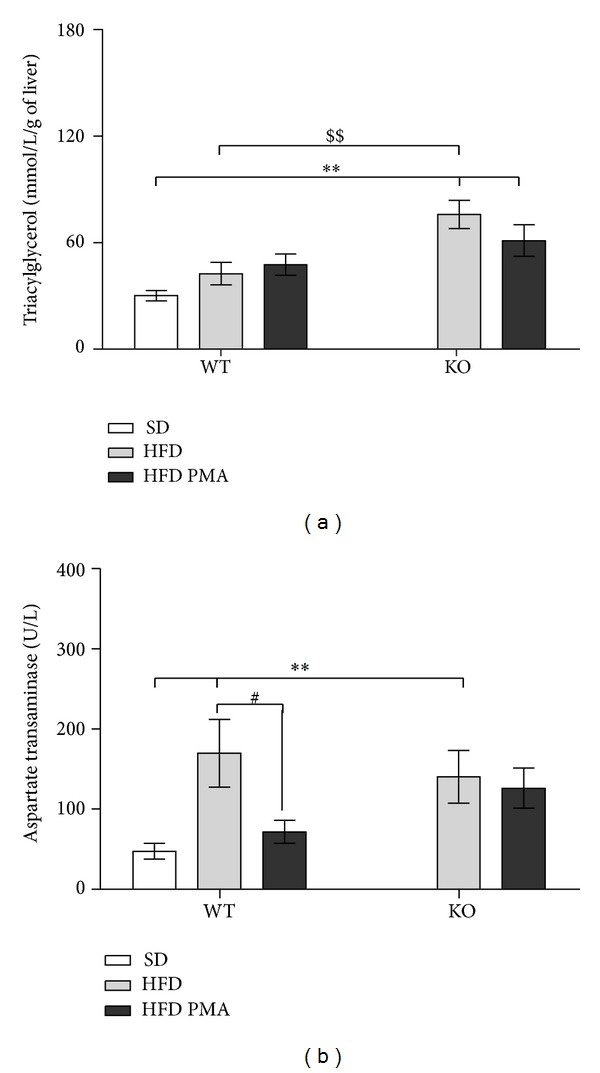
Triacylglycerol levels in the livers (a) and aspartate aminotransferase levels in the plasma (b) of WT mice fed with a standard diet (SD) or WT and PPAR*α*-knockout (KO) subjected to a high-fat diet and treated with oleic acid (HFD) or palmitoleic acid (HFD PMA). The data are presented as mean ± SEM. *P < 0.05 and **P < 0.01 versus WT SD; ^#^ *P* < 0.05 HFD versus HFD PMA; and ^$$^ *P* < 0.01 KO versus the respective WT control (one-way ANOVA followed by Bonferroni correction).

**Figure 3:**
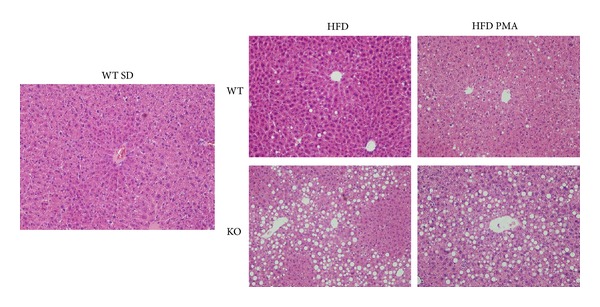
Histological slices of livers colored by hematoxylin and eosin (HE) at 40x magnification. Livers of WT mice fed with a standard diet (SD) or WT and PPAR*α*-knockout (KO) mice submitted to a high fat diet and treated with oleic acid (HFD) or palmitoleic acid (HFD PMA).

One of the tissues that was most affected by the HFD was the liver; indeed, the weight of the liver was markedly increased by the HFD in both WT and KO mice (Table 2); however, the HFD only promoted a significant increase in triacylglycerols in the livers of KO mice (Figure 2(a)), and we only observed severe steatosis in liver histological slices from KO mice fed with the HFD (Figure 3). Both WT and KO mice had an increase in the serum levels of the liver damage marker aspartate transaminase when subjected to the HFD (Figure 2(b)). PMA did not alter the weight of the liver or the ectopic accumulation of fat in the liver and did not seem to modulate hepatic steatosis, but this monounsaturated fatty acid almost completely restored the levels of aspartate transaminase in the serum of WT mice, indicating that there may be some protective effect of PMA on liver injury (Figures 2 and 3).

**Figure 4:**
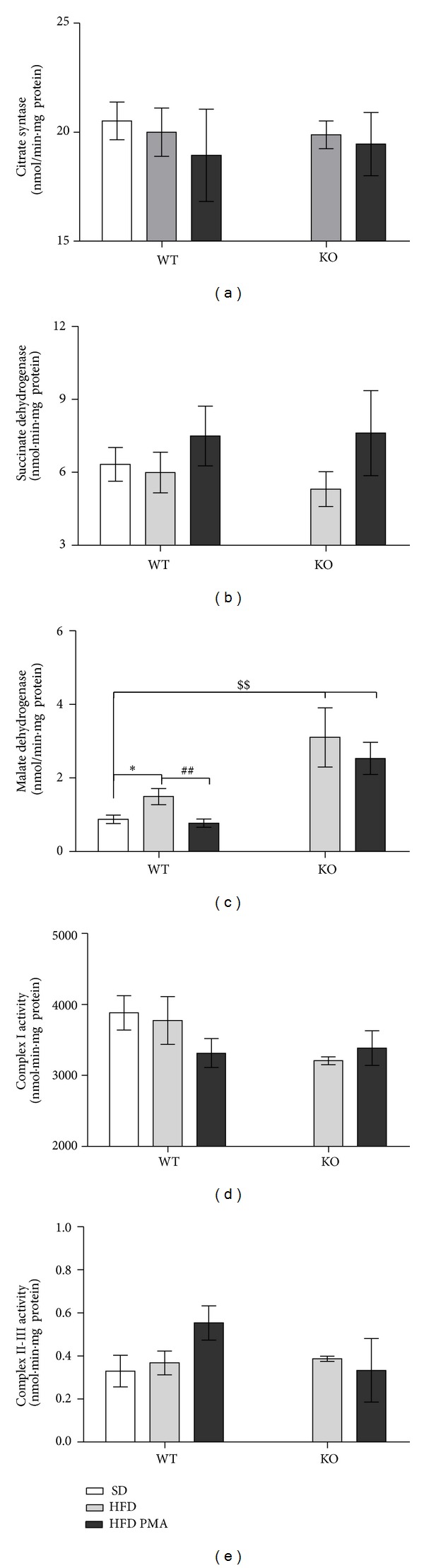
Activity of citrate synthase (a), succinate dehydrogenase (b), malate dehydrogenase (c), complex I (d), and complex II-III (e) in WT mice fed with standard diet (SD) or C57 and PPAR*α*-knockout (KO) mice fed with a high-fat diet and treated with oleic acid (HFD) or palmitoleic acid (HFD PMA). The data are presented as mean ± SEM. *P < 0.05 versus WT SD; ^##^ *P* < 0.01 HFD versus HFD PMA; and ^$$^ *P* < 0.05 KO versus the respective C57 control (one-way ANOVA followed by Bonferroni correction).

The activity of several Krebs cycle enzymes (citrate synthase (Figure 4(a)), succinate dehydrogenase (Figure 4(b)), and malate dehydrogenase (Figure 4(c))) and the activity of the electron transport chain complexes I and III (Figures 4(d) and 4(e)) were assessed. Surprisingly, of all these enzymes and complexes, the HFD only promoted a difference in the activity of malate dehydrogenase, and the effects of the HFD on the malate dehydrogenase activity of KO mice were even greater. In spite of this, supplementation with palmitoleic acid only decreased the activity of malate dehydrogenase in WT mice (Figure 4(c)).

**Figure 5:**
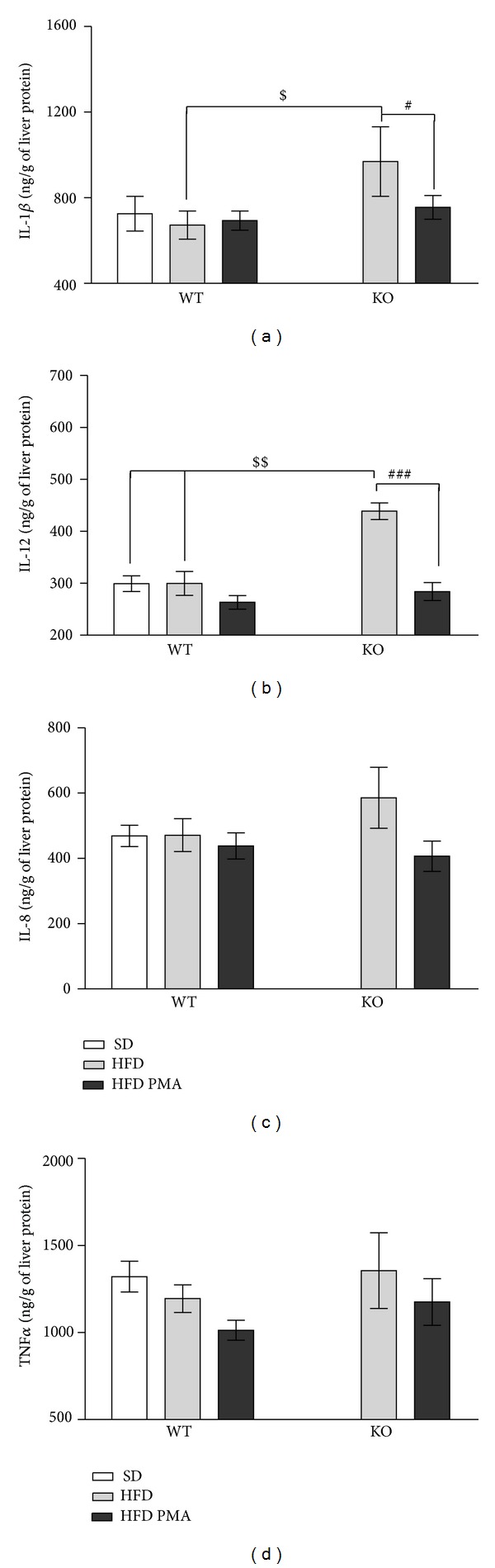
Hepatic levels of interleukin-1*β* (IL-1*β*) (a), interleukin-12 (IL-12) (b), interleukin-8 (IL-8) (c), and tumor necrosis factor-*α* (TNF*α*) (d) in WT mice fed with a standard diet (SD) or WT and PPAR*α*-knockout (KO) mice fed with a high-fat diet and treated with oleic acid (HFD) or palmitoleic acid (HFD PMA). The data are presented as mean ± SEM. ^#^ *P* < 0.05 and ^###^ *P* < 0.001 HFD versus HFD PMA; ^$^ *P* < 0.05 and ^$$^ *P* < 0.01 KO versus WT (one-way ANOVA followed by Bonferroni correction).

Surprisingly, the hepatic steatosis induced by the HFD was associated with liver inflammation in the KO mice but not in WT mice, as indicated by increased hepatic levels of IL-1*β* (Figure 5(a)), IL-12 (Figure 5(b)) and a trend toward higher levels of IL-8 (Figure 5(c)) and TNF-*α* (Figure 5(d)) in the livers of KO mice when compared to WT mice subjected to the SD. Furthermore, in KO mice, palmitoleate reduced the hepatic levels of IL1-*β* and IL-12 and caused a trend toward reduced levels of IL-8 and TNF-*α* in liver (Figure 5).

**Figure 6:**
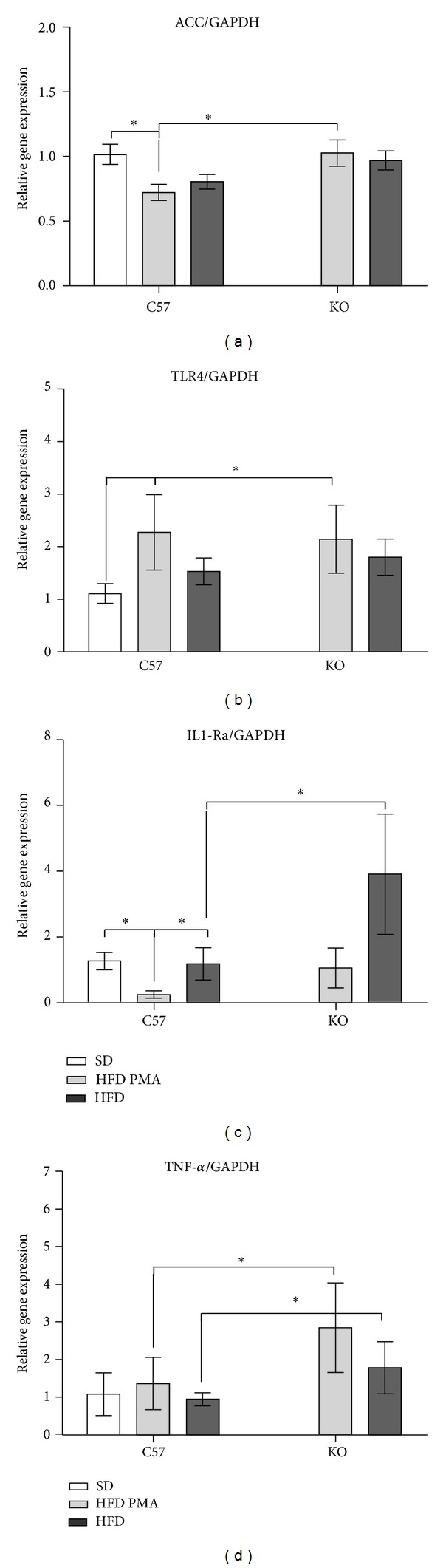
mRNA expression of acetyl-CoA carboxylase (ACC) (a), Toll-like receptor-4 (TLR-4) (b), antagonist receptor of interleukin-1 (IL-1Ra) (c), and tumor necrosis factor-*α* (TNF-*α*) (d) in WT mice fed with standard diet (SD) or WT and PPAR*α*-knockout (KO) mice fed with a high-fat diet and treated with oleic acid (HFD) or palmitoleic acid (HFD PMA). The data are presented as mean ± SEM. *P < 0.05 versus SD; ^#^ *P* < 0.05 HFD versus HFD PMA; and ^$^ *P* < 0.05 KO versus the respective WT control (one-way ANOVA followed by Bonferroni correction).

Although the HFD decreased the mRNA expression of ACC and IL-1Ra in WT mice (Figures 6(a) and 6(c)), the HFD upregulated the expression of TLR4 both in WT and KO mice (Figure 6(b)). In addition, the expression of TNF*α* was not modulated by the HFD but was increased only by the knockout of PPAR*α* (Figure 6(d)). Palmitoleate did not affect the expression of ACC mRNA (Figure 6(a)), but it tended to reduce the hepatic expression of TLR-4 and TNF*α* (Figures 6(b) and 6(d)), reversed the decrease in IL-1Ra mRNA expression in WT, and dramatically increased IL-1Ra mRNA expression in KO mice (Figure 6(c)).

**Figure 7:**
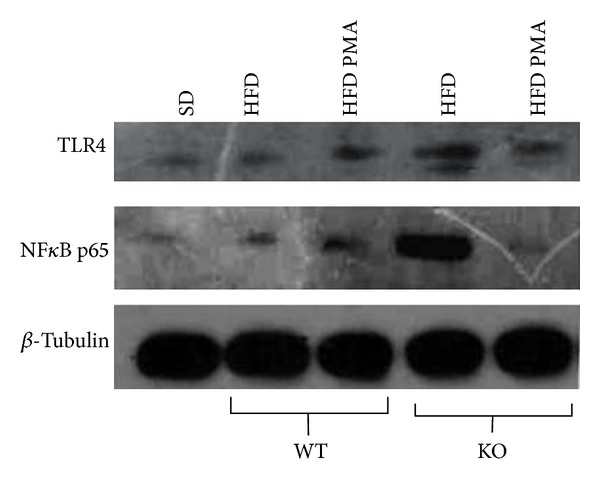
Protein levels of Toll-like receptor-4 (TLR-4) and serine phosphorylation of NF*Κ*B p65 in the liver of WT mice fed with a standard diet (SD) or WT and PPAR*α*-knockout (KO) mice fed with high-fat diet and treated with oleic acid (HFD) or palmitoleic acid (HFD PMA).

Along with increasing TLR4 mRNA levels, TLR4 protein levels and the phosphorylation of NF*κ*B (p65) in serine were increased in KO mice that were fed with the HFD (Figure 7); treatment with palmitoleic acid decreased both of these inflammatory mediators, reducing the levels of TLR4 and the phosphorylation of NF*Κ*B (p65) (Figure 7).

## 4. Discussion

In this study, we investigated the hypothesis that the nuclear receptor PPAR*α* may be involved in the beneficial activities of palmitoleic acid in attenuating the development of HFD-induced hepatic steatosis and inflammation and disruptions of glucose homeostasis. In contrast to this hypothesis, our main findings indicated that palmitoleic acid exerts its beneficial effects on hepatic inflammation and damage and improves insulin sensitivity through mechanisms that do not require PPAR*α*.

The activation of PPAR*α* seems to be a promising target in the treatment of NAFLD because PPAR*α* is also a key regulator of the genes involved in fatty acid oxidation [33–36] and anti-inflammatory effects [37, 38]. Indeed, it has been previously shown that the lack of PPAR*α* may cause an important hepatic steatosis and liver inflammation [34, 39]. In our study, we observed that the PPAR*α* KO mice had exacerbated hepatic steatosis and liver inflammation after 12 weeks of exposure to a HFD. Consistent with this, Su et al. (2014) showed that defects in PPAR*α* signaling induced mitochondrial and stress oxidative in mice fed with a high-fructose diet [40].

The effects of palmitoleic acid on NAFLD are controversial; while Guo et al. [12] showed that palmitoleic acid increased the deposition of fatty acids in the liver, Yang et al. [13] observed that it improved steatosis, reducing the ectopic deposition of triacylglycerols. In this work, we observed no effect of palmitoleic acid on steatosis or the ectopic deposition of triacylglycerols in liver caused by the HFD in both groups of mice, especially the KO mice, which had more pronounced NAFLD. The different effects of palmitoleic acid on NAFLD in these studies may be explained by the treatment times, dosages of PMA, and models used for the induction of steatosis.

We also observed other effects of palmitoleic acid, which is an important signaling molecule that is mainly produced by white adipose tissue and has been described as an insulin-sensitizing hormone that is capable of modulating several metabolic processes, such as glucose disposal and insulin sensitivity in skeletal muscle and lipids deposition in liver [11, 41]. Similarly, our findings indicate that palmitoleic acid increases the uptake of glucose in isolated muscles under stimulated conditions and improves the peripheral response to insulin in mice that are fed a HFD by mechanisms that are not regulated by PPAR*α*.

Lipogenesis in hepatocytes is under the control of a series of critical genes, such as sterol regulatory element binding protein-1 (SREBP1), fatty acid synthase (FAS), acetyl-CoA carboxylase enzymes (ACC), and stearoyl coenzyme A desaturase 1 (SCD-1). Of these, FAS and ACC seem to be particularly rate-limiting enzymes that are responsible for de novo lipogenesis, which may be increased in NAFLD [42, 43]. Concordantly, we observed that PPAR*α*-KO mice had increases in the expression of ACC mRNA with consequently exacerbated ectopic deposition of fat in the liver, whereas WT mice had lower expression of ACC, which indicated a balancing regulatory mechanism in these animals, most likely via the activation of PPAR*α*; this may explain why WT mice showed lower NAFLD and inflammation in response to the HFD. Combined with this increase in ACC, the hepatic levels of proinflammatory cytokines were increased in KO mice that were fed with the HFD.

The HFD increased the activity of malate dehydrogenase (MDH) in WT mice and promoted an even greater increase in the KO mice. A higher activity of this enzyme, which catalyzes the conversion of malate to oxaloacetate and the reverse reaction, could lead to an increase of malate levels in the mitochondria. Malate can be transported to the cytosol and converted by MDH to oxaloacetate, a precursor in the gluconeogenesis pathway [44, 45]. However, in addition to this increase in MDH activity, our group observed that PPAR*α* KO mice subjected to a HFD had lower gluconeogenesis in pyruvate tolerance test (data not shown), indicating that this excess of Krebs cycle intermediates may be converted to other pathways, such as de novo lipogenesis, as indicated by the higher expression levels of ACC and by the more severe steatosis observed in the KO mice. Palmitoleic acid did not modulate the activity of the Krebs cycle enzymes or the electron chain transporter.

Although palmitoleic acid did not improve hepatic steatosis, this *ω*-7 fatty acid reduced the serum aspartate transaminase levels in both WT and PPAR*α*-knockout mice, which indicates that the observed reduction in liver damage is independent of PPAR*α*. Previous studies have reported that higher levels of AST indicate a greater degree of inflammation in the liver [46]; we observed an increase in the hepatic levels of proinflammatory cytokines in PPAR*α*-knockout mice fed with the HFD, corroborating several studies [11, 14, 35, 39]. Surprisingly, palmitoleic acid diminishes the hepatic inflammation of these knockout mice, decreasing the levels of IL1-*β* and IL-12 in the liver, indicating once again that the beneficial effect of palmitoleic acid on the liver occurs by PPAR*α*-independent mechanisms.

It has been reported that saturated fatty acids such palmitic acid (C16:0) could increase inflammation through TLR4 activation [47]. Indeed, some authors have shown that HFD supplementation also increased the expression of TLR4, suggesting that this receptor played a main role in the development of NAFLD [48, 49]. In the present study, we observed that HFD increased the expression of TLR4 mRNA in both WT and KO mice and increased TLR4 protein levels in the livers of KO mice, but palmitoleic acid reduced the hepatic mRNA and protein levels of TLR4, suggesting that this monounsaturated fatty acid has a strong anti-inflammatory effect.

The activation of PPAR*α* has been described as an inhibitory mechanism for NF*κ*B activation, consequently reducing the expression of proinflammatory genes and the production of proinflammatory cytokines, such TNF-*α* and IL-12 [50]. Consistent with this, we observed that the expression of TNF-*α* was increased in KO mice even when subjected to an SD. However because palmitoleic acid was capable of decreasing the serine phosphorylation of NF*κ*B p65, which was increased by the HFD only in PPAR*α* KO mice, we propose that the anti-inflammatory effect of palmitoleic acid involves other mechanisms for the inhibition of NF*κ*B than the activation of PPAR*α*. Indeed, the HFD decreased the expression of IL-1Ra in both genotypes, while palmitoleic acid increased the expression of IL-1Ra in both WT and KO mice. However, the expression of IL-1Ra was increased to a greater extent in KO mice that were subjected to the HFD and treated with palmitoleic acid. Other studies have shown that AMPK activation can elevate IL-1Ra levels independently of PPAR*α* activation [51].

Therefore, we conclude that supplementation with palmitoleic acid but not with oleic acid can attenuate insulin resistance, liver damage, and inflammation induced by a HFD via mechanisms that do not depend on PPAR*α*.

## Supplementary Material

Effects of different genotypes about peripheral parameters. Body weight (BW), tissues weight and serum levels of triacylglycerol, total cholesterol, HDL, estimate LDL, fasting glycemia, aspartate transaminase and triacylglycerol levels in liver of wild type (WT) and PPAR*α* knockout (KO) mice fed with a standard diet (SD).

## Figures and Tables

**Table 1:** Sequences of forward and reverse primers used for qRT-PCR.

Gene	Forward primer	Reverse primer
GAPDH	CAAGCTCATTTCCTGGTATGACA	GCCTCTCTTGCTCAGTGTCC
ACC	CCAGCAGATTGCCAACATC	ACTTCGGTACCTCTGCACCA
TLR4	TCCAGCCACTGAAGTTCT	CAGCAAAGTCCCTGATGA
TNF-*α*	TCTACTGAACTTCGGGGTGA	GATCTGAGTGTGAGGGTCTGG
IL-1Ra	GCAAGATGCAAGCCTTCAGA	CCTTGTAAGTACCCAGCAATGA

**Table 2:** Body weight (BW); tissue masses; and plasma levels of triacylglycerol, total cholesterol, HDL, estimated LDL, and fasting glycemia in wild-type (WT) and PPAR*α*-knockout (KO) mice fed with a standard diet (SD) or a high fat diet (HFD) and either untreated or treated with oleic acid (HFD) or palmitoleic acid (HFD PMA).

	WT mice			PPAR*α*-KO mice	
	SD	HFD	HFD PMA	HFD	HFD PMA
Initial BW (g)	26.33 ± 0.45 (*n* = 6)	26.87 ± 0.73 (*n* = 6)	27.2 ± 0.62 (*n* = 6)	26.71 ± 0.61 (*n* = 4)	25.73 ± 0.62 (*n* = 5)

10 wk BW (g)	29.80 ± 1.04 (*n* = 6)	39.83 ± 1.90* (*n* = 6)	39.39 ± 1.68* (*n* = 6)	34.80 ± 1.26* (*n* = 4)	33.98 ± 1.72* (*n* = 5)

Final BW (g)	28.66 ± 0.73 (*n* = 6)	38.82 ± 1.90* (*n* = 6)	38.4 ± 1.71* (*n* = 6)	33.39 ± 1.09* (*n* = 4)	32.47 ± 1.69* (*n* = 5)

Adiposity index (g)	1.33 ± 0.20 (*n* = 6)	3.51 ± 0.42* (*n* = 5)	3.82 ± 0.42* (*n* = 6)	2.41 ± 0.28^$^ (*n* = 4)	2.19 ± 0.40^$^ (*n* = 5)

Brown adipose tissue weight (g)	0.42 ± 0.05 (*n* = 6)	0.45 ± 0.04 (*n* = 5)	0.45 ± 0.07 (*n* = 6)	0.32 ± 0.03 (*n* = 4)	0.33 ± 0.04 (*n* = 5)

Liver weight (g)	1.05 ± 0.02 (*n* = 6)	1.19 ± 0.05* (*n* = 5)	1.19 ± 0.06* (*n* = 6)	1.26 ± 0.05* (*n* = 4)	1.23 ± 0.08* (*n* = 5)

Triacylglycerol (mg/dL)	75.39 ± 5.37 (*n* = 6)	87.86 ± 10.44 (*n* = 4)	88.25 ± 7.96 (*n* = 6)	94.28 ± 5.23 (*n* = 4)	91.05 ± 2.75 (*n* = 5)

Total cholesterol (mg/dL)	133.9 ± 7.18 (*n* = 6)	177.5 ± 8.68* (*n* = 5)	173.7 ± 4.45* (*n* = 6)	193.4 ± 19.99* (*n* = 4)	187.3 ± 14.64* (*n* = 5)

HDL cholesterol (mg/dL)	109.7 ± 5.32 (*n* = 6)	152.8 ± 12.14* (*n* = 5)	145.7 ± 12.54 (*n* = 6)	123.2 ± 7.65^$^ (*n* = 4)	111.4 ± 8.79^$^ (*n* = 5)

Estimated LDL (mg/dL)	21.28 ± 4.25 (*n* = 4)	20.72 ± 5.32 (*n* = 4)	31.34 ± 2.99 (*n* = 4)	51.37 ± 17.30^$^ (*n* = 4)	47.23 ± 10.88^$^ (*n* = 4)

Fasting glucose (mg/dL)	130.1 ± 13.66 (*n* = 6)	171.3 ± 9.58* (*n* = 6)	150.0 ± 10.25^#^ (*n* = 6)	124.7 ± 8.38^∗$$$^ (*n* = 4)	98.0 ± 4.41^#$$$^ (*n* = 5)

The data are presented as mean ± SEM. *P < 0.05 versus WT SD; ^#^ *P* < 0.05 HFD versus HFD PMA; and ^$^ *P* < 0.05 and ^$$$^ *P* < 0.001 KO versus the respective WT control (one-way ANOVA followed by Bonferroni correction).

## References

[1] Zelber-Sagi S, Nitzan-Kaluski D, Halpern Z, Oren R (2006). Prevalence of primary non-alcoholic fatty liver disease in a population-based study and its association with biochemical and anthropometric measures. *Liver International*.

[2] Hotamisligil GS, Erbay E (2008). Nutrient sensing and inflammation in metabolic diseases. *Nature Reviews Immunology*.

[3] Wang YC, McPherson K, Marsh T, Gortmaker SL, Brown M (2011). Health and economic burden of the projected obesity trends in the USA and the UK. *The Lancet*.

[4] Makki K, Froguel P, Wolowczuk I (2013). Adipose tissue in obesity-related inflammation and insulin resistance: cells, cytokines, and chemokines. *ISRN Inflammation*.

[5] Singer K, Eng DS, Lumeng CN, Gebremariam A, Lee JM (2014). The relationship between body fat mass percentiles and inflammation in children. *Obesity*.

[6] Chen L, Magliano DJ, Zimmet PZ (2012). The worldwide epidemiology of type 2 diabetes mellitus—present and future perspectives. *Nature Reviews Endocrinology*.

[7] Starley BQ, Calcagno CJ, Harrison SA (2010). Nonalcoholic fatty liver disease and hepatocellular carcinoma: a weighty connection. *Hepatology*.

[8] Pawella LM, Hashani M, Eiteneuer E (2014). Perilipin discerns chronic from acute hepatocellular steatosis. *Journal of Hepatology*.

[9] Leverve X (2003). Hyperglycemia and oxidative stress: complex relationships with attractive prospects. *Intensive Care Medicine*.

[10] Sanz M, Sánchez-Martín C, Detaille D (2011). Acute mitochondrial actions of glitazones on the liver: a crucial parameter for their antidiabetic properties. *Cellular Physiology and Biochemistry*.

[11] Cao H, Gerhold K, Mayers JR, Wiest MM, Watkins SM, Hotamisligil GS (2008). Identification of a lipokine , a lipid hormone linking adipose tissue to systemic metabolism. *Cell*.

[12] Guo X, Li H, Xu H (2012). Palmitoleate induces hepatic steatosis but suppresses liver inflammatory response in mice. *PLoS ONE*.

[13] Yang ZH, Miyahara H, Hatanaka A (2011). Chronic administration of palmitoleic acid reduces insulin resistance and hepatic lipid accumulation in KK-Ay mice with genetic type 2 diabetes. *Lipids in Health and Disease*.

[14] Bolsoni-Lopes A, Festuccia WT, Farias TS (2013). Palmitoleic acid (n-7) increases white adipocyte lipolysis and lipase content in a PPAR*α*-dependent manner. *The American Journal of Physiology—Endocrinology and Metabolism*.

[15] Cherkaoui-Malki M, Surapureddi S, El Hajj HI, Vamecq J, Andreoletti P (2012). Hepatic steatosis and peroxisomal fatty acid beta-oxidation. *Current Drug Metabolism*.

[16] Daynes RA, Jones DC (2002). Emerging roles of PPARs in inflammation and immunity. *Nature Reviews Immunology*.

[17] Reeves PG, Nielsen FH, Fahey GC (1993). AIN-93 purified diets for laboratory rodents: final report of the American Institute of Nutrition ad hoc writing committee on the reformulation of the AIN-76A rodent diet. *Journal of Nutrition*.

[18] Srisawasdi P, Chaloeysup S, Teerajetgul Y (2011). Estimation of plasma small dense LDL cholesterol from classic lipid measures. *American Journal of Clinical Pathology*.

[19] Yin Y, Yu Z, Xia M, Luo X, Lu X, Ling W (2012). Vitamin D attenuates high fat diet-induced hepatic steatosis in rats by modulating lipid metabolism. *European Journal of Clinical Investigation*.

[20] Folch J, Lees M, Sloane Stanley GH (1957). A simple method for the isolation and purification of total lipides from animal tissues. *The Journal of Biological Chemistry*.

[21] Bonora E, Moghetti P, Zancanaro C (1989). Estimates of in vivo insulin action in man: comparison of insulin tolerance tests with euglycemic and hyperglycemic glucose clamp studies. *Journal of Clinical Endocrinology and Metabolism*.

[22] Bergmeyer H, Bernet E, Bergmeyer H (1974). Determination of glucose with glucoseoxidase and peroxidase. *Methods of Enzymatic Analysis*.

[23] Challiss RAJ, Lozeman FJ, Leighton B, Newsholme EA (1986). Effects of the *β*-adrenoceptor agonist isoprenaline on insulin-sensitivity in soleus muscle of the rat. *Biochemical Journal*.

[24] Espinal J, Dohm GL, Newsholme EA (1983). Sensitivity to insulin of glycolysis and glycogen synthesis of isolated soleus-muscle strips from sedentary, exercised and exercise-trained rats. *Biochemical Journal*.

[25] Leighton B, Budohoski L, Lozeman FJ, Challiss RA, Newsholme EA (1985). The effect of prostaglandins E1, E2 and F2 alpha and indomethacin on the sensitivity of glycolysis and glycogen synthesis to insulin in stripped soleus muscles of the rat. *The Biochemical journal*.

[26] Lowry OH, Rosebrough NJ, Farr AL, Randall RJ (1951). Protein measurement with the folin phenol reagent. *The Journal of biological chemistry*.

[27] Srere PA (1974). Controls of citrate synthase activity. *Life Sciences*.

[28] Fischer JC, Ruitenbeek W, Berden JA (1985). Differential investigation of the capacity of succinate oxidation in human skeletal muscle. *Clinica Chimica Acta*.

[29] Kitto GB, Wassarman PM, Kaplan NO (1966). Enzymatically active conformers of mitochondrial malate dehydrogenase. *Proceedings of the National Academy of Sciences of the United States of America*.

[30] Cassina A, Radi R (1996). Differential inhibitory action of nitric oxide and peroxynitrite on mitochondrial electron transport. *Archives of Biochemistry and Biophysics*.

[31] Chomczynski P, Sacchi N (1987). Single-step method of RNA isolation by acid guanidinium thiocyanate-phenol-chloroform extraction. *Analytical Biochemistry*.

[32] Livak KJ, Schmittgen TD (2001). Analysis of relative gene expression data using real-time quantitative PCR and the 2^-ΔΔC^T method. *Methods*.

[33] Mandard S, Müller M, Kersten S (2004). Peroxisome proliferator-activated receptor *α* target genes. *Cellular and Molecular Life Sciences*.

[34] Stienstra R, Mandard S, Patsouris D, Maass C, Kersten S, Müller M (2007). Peroxisome proliferator-activated receptor *α* protects against obesity-induced hepatic inflammation. *Endocrinology*.

[35] Monsalve FA, Pyarasani RD, Delgado-Lopez F, Moore-Carrasco R (2013). Peroxisome proliferator-activated receptor targets for the treatment of metabolic diseases. *Mediators of Inflammation*.

[36] Ip E, Farrell G, Hall P, Robertson G, Leclercq I (2004). Administration of the potent ppar*α* agonist, Wy-14,643, reverses nutritional fibrosis and steatohepatitis in mice. *Hepatology*.

[37] Tapia G, Valenzuela R, Espinosa A (2014). N-3 long-chain PUFA supplementation prevents high fat diet induced mouse liver steatosis and inflammation in relation to PPAR-*α* upregulation and NF-*κ*B DNA binding abrogation. *Molecular Nutrition & Food Research*.

[38] Quintero P, Arrese M (2013). Nuclear control of inflammation and fibrosis in nonalcoholic steatohepatitis : therapeutic potential of dual peroxisome proliferator-activated receptor alpha/delta agonism. *Hepatology*.

[39] Hashimoto T, Fujita T, Usuda N (1999). Peroxisomal and mitochondrial fatty acid *β*-oxidation in mice nullizygous for both peroxisome proliferator-activated receptor and peroxisomal fatty acyl-CoA oxidase: Genotype correlation with fatty liver phenotype. *Journal of Biological Chemistry*.

[40] Su Q, Baker C, Christian P (2014). Hepatic mitochondrial and ER stress induced by defective PPAR*α* signaling in the pathogenesis of hepatic steatosis. *American Journal of Physiology. Endocrinology and Metabolism*.

[41] Dimopoulos N, Watson M, Sakamoto K, Hundal HS (2006). Differential effects of palmitate and palmitoleate on insulin action and glucose utilization in rat L6 skeletal muscle cells. *Biochemical Journal*.

[42] Koo SH (2013). Nonalcoholic fatty liver disease: molecular mechanisms for the hepatic steatosis. *Clinical and Molecular Hepatology*.

[43] Nakamuta M, Kohjima M, Morizono S (2005). Evaluation of fatty acid metabolism-related gene expression in nonalcoholic fatty liver disease. *International Journal of Molecular Medicine*.

[44] Jitrapakdee S, Wutthisathapornchai A, Wallace JC, MacDonald MJ (2010). Regulation of insulin secretion: role of mitochondrial signalling. *Diabetologia*.

[45] Rolo AP, Teodoro JS, Palmeira CM (2012). Role of oxidative stress in the pathogenesis of nonalcoholic steatohepatitis. *Free Radical Biology and Medicine*.

[46] Sharifov OF, Xu X, Gaggar A (2013). Anti-inflammatory mechanisms of apolipoprotein a-i mimetic peptide in acute respiratory distress syndrome secondary to sepsis. *PLoS ONE*.

[47] Huang S, Rutkowsky JM, Snodgrass RG (2012). Saturated fatty acids activate TLR-mediated proinflammatory signaling pathways. *Journal of Lipid Research*.

[48] Roh YS, Seki E (2013). Toll-like receptors in alcoholic liver disease, non-alcoholic steatohepatitis and carcinogenesis. *Journal of Gastroenterology and Hepatology*.

[49] le Roy T, Llopis M, Lepage P (2013). Intestinal microbiota determines development of non-alcoholic fatty liver disease in mice. *Gut*.

[50] Genolet R, Wahli W, Michalik L (2004). PPARs as drug targets to modulate inflammatory responses?. *Current Drug Targets: Inflammation and Allergy*.

[51] Tomizawa A, Hattori Y, Inoue T, Hattori S, Kasai K (2011). Fenofibrate suppresses microvascular inflammation and apoptosis through adenosine monophosphate-activated protein kinase activation. *Metabolism: Clinical and Experimental*.

