# Supplementary material for: Palmitoleic Acid (N-7) Attenuates the Immunometabolic Disturbances Caused by a High-Fat Diet Independently of PPARα
Source: Mediators Inflamm. 2014 Jul 24;2014:582197. doi: 10.1155/2014/582197 (PMC4131426; doi:10.1155/2014/582197)
Supplement: Supplementary file 1 — Effects of different genotypes about peripheral parameters. Body weight (BW), tissues weight and serum levels of triacylglycerol, total cholesterol, HDL, estimate LDL, fasting glycemia, aspartate transaminase and triacylglycerol levels in liver of wild type (WT) and PPARα knockout (KO) mice fed with a standard diet (SD). [file 582197.f1.pdf]

SUPPLEMENTARY RESULTS

Table S1. Effects of different genotypes about peripheral parameters. Body weight (BW), tissues weight and serum levels of triacylglycerol, total cholesterol, HDL, estimate LDL, fasting glycemia, aspartate transaminase and triacylglycerol levels in liver of wild type (WT) and PPARα knockout (KO) mice fed with a standard diet (SD).

	WT SD			KO SD		
	Mean	SEM	n	Mean	SEM	n
Inicial body weight (g)	27,04	0,45	6	26,14	0,86	4
Final body weight (g)	28,66	0,73	6	28,70	0,96	4
Adiposity index (g)	1,33	0,20	6	1,15	0,19	4
Brown adipose tissue weight (g)	0,42	0,05	6	0,41	0,06	4
Liver weight (g)	1,05	0,03	6	1,06	0,06	4
Triacylglycerol (mg/dL)	87,86	10,44	4	94,29	5,23	4
Total cholesterol (mg/dL)	133,93	7,18	6	160,41	7,15	4
HDL cholesterol (mg/dL)	109,76	5,33	6	117,857*	8,84	4
Estimate LDL (mg/dL)	21,28	4,25	4	21,03	5,77	4
Fasting glucose (mg/dL)	130,17	13,67	6	92,50	14,89	4
AST (mg/dL)	47,54	9,75	4	48,30	6,18	4
Liver Triacylglycerol (mmol/L/g)	30,17	2,87	6	38,86	8,86	4

Data are presented as mean ± SEM. *p<0.05 WT SD *vs.* KO SD (T Test).
